# Dynamic Population on Bio-Inspired Algorithms Using Machine Learning for Global Optimization

**DOI:** 10.3390/biomimetics9010007

**Published:** 2023-12-25

**Authors:** Nicolás Caselli, Ricardo Soto, Broderick Crawford, Sergio Valdivia, Elizabeth Chicata, Rodrigo Olivares

**Affiliations:** 1 Escuela de Ingeniería Informática, Pontificia Universidad Católica de Valparaíso, Valparaíso 2362807, Chile; broderick.crawford@pucv.cl (B.C.); elizabeth.chicata.c@mail.pucv.cl (E.C.); 2 Departamento de Tecnologías de Información y Comunicación, Universidad de Valparaíso, Valparaíso 2361864, Chile; sergio.valdivia@uv.cl; 3 Escuela de Ingeniería Informática, Universidad de Valparaíso, Valparaíso 2362905, Chile; rodrigo.olivares@uv.cl

**Keywords:** autonomous algorithms, metaheuristics, high-density functions, optimization, continuous population, CEC benchmark, particle swarm optimization, cuckoo search algorithm, bat algorithm, performance comparison

## Abstract

In the optimization field, the ability to efficiently tackle complex and high-dimensional problems remains a persistent challenge. Metaheuristic algorithms, with a particular emphasis on their autonomous variants, are emerging as promising tools to overcome this challenge. The term “autonomous” refers to these variants’ ability to dynamically adjust certain parameters based on their own outcomes, without external intervention. The objective is to leverage the advantages and characteristics of an unsupervised machine learning clustering technique to configure the population parameter with autonomous behavior, and emphasize how we incorporate the characteristics of search space clustering to enhance the intensification and diversification of the metaheuristic. This allows dynamic adjustments based on its own outcomes, whether by increasing or decreasing the population in response to the need for diversification or intensification of solutions. In this manner, it aims to imbue the metaheuristic with features for a broader search of solutions that can yield superior results. This study provides an in-depth examination of autonomous metaheuristic algorithms, including Autonomous Particle Swarm Optimization, Autonomous Cuckoo Search Algorithm, and Autonomous Bat Algorithm. We submit these algorithms to a thorough evaluation against their original counterparts using high-density functions from the well-known CEC LSGO benchmark suite. Quantitative results revealed performance enhancements in the autonomous versions, with Autonomous Particle Swarm Optimization consistently outperforming its peers in achieving optimal minimum values. Autonomous Cuckoo Search Algorithm and Autonomous Bat Algorithm also demonstrated noteworthy advancements over their traditional counterparts. A salient feature of these algorithms is the continuous nature of their population, which significantly bolsters their capability to navigate complex and high-dimensional search spaces. However, like all methodologies, there were challenges in ensuring consistent performance across all test scenarios. The intrinsic adaptability and autonomous decision making embedded within these algorithms herald a new era of optimization tools suited for complex real-world challenges. In sum, this research accentuates the potential of autonomous metaheuristics in the optimization arena, laying the groundwork for their expanded application across diverse challenges and domains. We recommend further explorations and adaptations of these autonomous algorithms to fully harness their potential.

## 1. Introduction

In operation research fields, the role of optimization algorithms is undeniably pivotal. As the complexities of problems across various domains burgeon, so does the necessity for sophisticated optimization strategies [1,2]. Metaheuristics, with their inherent capacity to explore vast solution spaces, have risen to prominence [3]. Yet, with the increasing dimensionality and intricacy of problems, the traditional static management of solution populations often proves inadequate [4,5].

A significant challenge that modern optimization algorithms face is dynamically managing their populations of solutions [6,7]. Traditional approaches often employ static populations, which, while simpler to implement and manage, frequently fall short in adapting to the evolving nature of complex optimization landscapes [8]. Such static management can lead to premature convergence, where the algorithm becomes stuck in local optima without fully exploring potential solutions [9]. Furthermore, without a dynamic adaptation mechanism, algorithms might not efficiently exploit promising regions or explore lesser-known areas of the solution space. This stagnation not only diminishes the algorithm’s potential to locate the global optimum but also curtails its versatility across varied problem instances [10].

In the modern landscape of optimization, the dynamic management of solution populations has transitioned from being a mere enhancement to an absolute necessity [11,12]. Addressing this critical need, our study sets forth with a meticulously crafted approach that marries the strengths of both metaheuristics and clustering techniques. At the forefront of our strategy are three distinguished optimization metaheuristics: particle swarm optimization (PSO), cuckoo search algorithm (CSA), and bat algorithm (BA). Their selection is predicated on their consistent performance and resilience across diverse optimization challenges. Their legacy of success, coupled with their inherent capabilities of exploration and exploitation, underscores their suitability for our ambitious undertaking [11]. To elevate the potential of these bio-inspired solvers, we incorporate the ability of the Density-Based Spatial Clustering of Applications with Noise (DBSCAN) algorithm, a wide-known clustering methodology by the scientific community [13,14]. DBSCAN’s reputation is anchored in its exceptional ability to discern and classify clusters with varied shapes and densities, an aspect where many clustering paradigms falter [15]. Our proposition leans on this strength of DBSCAN, envisaging a harmonious collaboration that magnifies the dynamism in managing solution populations. This union is anticipated to dynamically manage and categorize populations of continuous solutions, thereby refining the adaptability and efficiency of the metaheuristics. The contribution of our study focuses on applying logic centered on the different clusters provided by DBSCAN. Depending on the characteristics of the solutions in each subset, we will apply an increase or decrease in the population in an autonomous way [16]. This variability in the population will enhance the metaheuristic’s ability to intensify or diversify solutions. To ensure the robustness and validity of our integrated approach, a rigorous and methodical evaluation is paramount. We have, therefore, chosen the harder functions of the CEC LSGO suite [17] as the testing ground for our proposition. These functions, renowned in the optimization community, embody a myriad of challenges, from multi-modality to shifting landscapes, serving as an ideal crucible to truly assess the mettle of our strategy [18,19,20]. the CEC LSGO suite, with its diverse and demanding function set, offers a comprehensive canvas, enabling us to probe the strengths and potential limitations of our approach under varied conditions. Our methodology for this evaluation will be meticulous, encompassing multiple runs, diverse initial conditions, and thorough statistical analyses.

The rest of the manuscript is described as follows: Section 2 presents the related work. Section 3 exposes SWEVOH: Self-adaptive Swarm Evolutionary Hybrid Algorithm. Experimental results are described in Section 4. At the end of the manuscript, conclusions are presented in Section 5.

## 2. Related Work

Optimization and metaheuristics stand at the forefront of innovative problem-solving across diverse fields, marking an era of significant evolution from traditional approaches to sophisticated strategies empowered by genetic algorithms and deep learning [21,22]. This relentless progression towards precision and efficiency has dramatically expanded the potential for discovering optimal solutions in complex landscapes [23,24,25,26].

Genetic and evolutionary algorithms have seen substantial refinements, with more sophisticated selection and mutation processes specifically tailored to tackle the challenges inherent in optimizing complex, high-dimensional systems [22,24]. This has led to algorithms that not only navigate but also effectively map the increasingly complex solution spaces [23,24,25]. Swarm-based optimization, notably particle swarm and ant colony optimization, has similarly advanced, now boasting enhanced capabilities for identifying global optima and skirting local optima—critical features in dynamic and unpredictable environments [27,28,29,30].

In the realm of metaheuristics with dynamic population management, several studies have addressed diverse optimization challenges. One study delves into an NP-hard multi-period production distribution problem, employing a memetic algorithm with population management to simultaneously handle production and distribution decisions, achieving significant savings compared to two-phase methods [31]. Another investigation focuses on a dynamic prey–predator spatial model, introducing the African buffalo optimization metaheuristic and employing autonomous multi-agents to regulate buffalo populations, achieving a balanced coexistence of prey and predators [32].

Other population-based approaches are explored in a study that designs a hybrid architecture, the Linear Modular Population Balancer, dynamically balancing and controlling population size based on learning components, demonstrating effectiveness across discrete and continuous optimization problems [33]. Feature selection, a challenging problem, is addressed using the Grasshopper Optimization Algorithm (GOA) with Evolutionary Population Dynamics to mitigate convergence and stagnation drawbacks, revealing superior performance on various datasets [34]. the Black Hole Algorithm is introduced as a nature-inspired optimization algorithm for data clustering, and a multi-population version is proposed, exhibiting precise results and high convergence rates on benchmark functions and real datasets [35].

Lastly, the Equilibrium Optimizer, inspired by dynamic mass balance, is enhanced with opposition-based learning, Lévy flight, and evolutionary population dynamics, resulting in EOOBLE, a competitive algorithm for high-dimensional global optimization problems, outperforming other metaheuristic algorithms [36].

Merging machine learning with conventional optimization techniques has catalyzed the creation of adaptive systems that continually learn and improve, endowing them with a level of autonomy that drastically enhances their effectiveness in complex process optimization [24,37]. Reinforcement learning and deep neural networks stand out as transformative tools, honing the search for solutions and propelling forward the frontiers of automation and predictive analytics [37,38,39]. the integration of diverse optimization strategies through hybridization has been a leap forward, yielding robust and efficient solutions [40,41,42]. Such integrative approaches have harnessed the adaptability of metaheuristics with the predictive power of machine learning to produce systems that dynamically adjust their search mechanisms, enhancing problem-solving in real-time. This fusion has not only improved algorithmic efficiency but has also broadened the scope of their application, enabling the tackling of previously elusive problems and adeptly handling the uncertainties and complexities of real-world systems [25,30,43].

The optimization landscape has been further enriched by the development of adaptive algorithms that fluidly transition between global and local search strategies, offering more effective exploration and exploitation of the solution space [3]. These adaptive methodologies have shown great promise in managing the variability and complexity of contemporary systems, finding applications in as diverse fields as logistics, energy system management, and the design of cutting-edge materials [23,24,29,39].

The convergence of optimization and machine learning continues to be a fertile area for research and development, heralding an era of intelligent optimization solutions that are not only faster and more efficient but also capable of adapting to an array of complex challenges [29,44,45]. These solutions are set to redefine the future of decision making and system analysis, providing innovative responses to the dynamic and ever-evolving environments of the modern world [25,30,37,39,43].

## 3. SWEVOH: Self-Adaptive Swarm Evolutionary Hybrid Algorithm

To adequately depict and elucidate the operation of our proposition, it is imperative to initiate by delineating the working principles of the metaheuristics chosen for our investigation. Consequently, we shall commence by providing detailed descriptions of these methodologies.

### 3.1. Metaheuristics

In this section, we will show which are the metaheuristics used to implement our working idea, which are their characteristics, their parameters, and a brief description of their behavior through a pseudo-code.

#### 3.1.1. Cuckoo Search Algorithm

Skilled in applying metaheuristics for diverse problem-solving, especially adept at large-scale combinatorial optimization within acceptable timeframes. Note that optimal solutions are not always guaranteed [46].

Nowadays. A full set of all nature-inspired algorithms can be found in [47], one of them is CSA, which has several study cases. CSA [48] is inspired by the obligate brood parasitism of some cuckoo species by laying their eggs in the nests of other bird species. In order to simplify the description of the CSA steps are described below:

1. Each cuckoo lays an egg at a time and drops it into a randomly selected nest.
2. The best nests with high-quality eggs will be carried over to the next generation.
3. The number of available host nests is fixed, and the egg laid by a cuckoo is discovered by the host bird with a probability $p_{a}\in \left[0,1\right]$ . In this case, a new random solution is generated.

Every new generation is determined b Equation (1).

(1) $$x_{i}^{d}\left(t+1\right)=x_{i}^{d}+\alpha Levy\left(\beta \right),\;\forall i\in \left\{1,\ldots ,m\right\}\wedge \forall d\in \left\{1,\ldots ,n\right\}$$

where $x_{i}^{d}$ is the element *d* of the solution vector *i* at iteration *t*. $x_{i}^{d}\left(t+1\right)$ is a solution in the iteration t+1 . α>0 is the step size which should be related to the scales of the problem of interest, the upper and lower bounds that the problem needs to be determined. Lévy flight is computed by Equation (2):

(2) $$Levy∼u=t^{\beta },\left(0<\beta <3\right)$$

Lévy flight involves random walks with infinite variance. Algorithm 1 includes pseudo-code for better understanding.

#### 3.1.2. Bat Algorithm

The bat algorithm is a metaheuristic optimization method based on microbats’ echolocation behavior, introduced by Yang in 2010 [49]. It has found wide application in various fields. the algorithm is inspired by the hunting behavior of bats and operates as follows:

- Initialization: BA starts with a population of bats, each representing a solution in the search space. Each bat has an initial position.
- Update: Each iteration involves bats updating positions through random flights and adjusting towards better solutions.
- Solution Improvement: If a bat finds a better solution, it updates its position. If it discovers a better global solution, the best global solution is also updated.
- Stopping Criterion: The algorithm iterates until a stopping criterion, like a maximum number of iterations, is met. Our three criteria are described as follows:
  1. All bats use echolocation to sense distance, and they also “know” the difference between food/prey and background barriers in some magical way.
  2. Bats fly randomly with velocity $v_{i}$ at position $x_{i}$ with a fixed frequency $f_{m}in$ , varying wavelength λ , and loudness $A_{0}$ to search for prey. They can automatically adjust the wavelength (or frequency) of their emitted pulses and adjust the rate of pulse emission r∈[0,1] , depending on the proximity of their target.
  3. Although the loudness can vary in many ways, we assume that the loudness varies from a large (positive) $A_{0}$ to a minimum constant value $A_{min}$

**Algorithm 1:** Pseudocode for Cuckoo Search
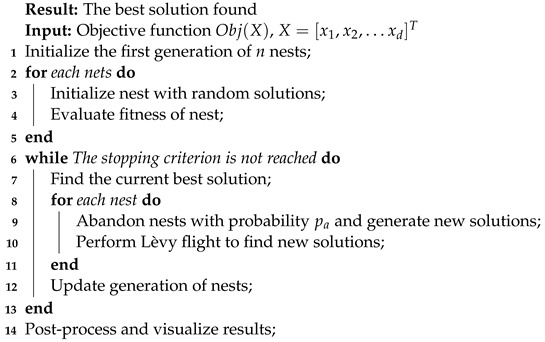


In addition, for simplicity, they also use the following approximations: in general, the frequency *f* in a range $\left[f_{min},f_{max}\right]$ corresponds to a range of wavelengths $\left[\lambda_{min},\lambda_{max}\right]$ . In fact, they just vary in frequency while fixed in the wavelength λ and assume $f\in [0,f_{max}]$ in their implementation. This is because λ and *f* are related due to the fact that λf=v is constant.

In simulations, they use virtual bats naturally to define the updated rules of their positions $x_{i}$ and velocities $v_{i}$ in a D-dimensional search space. the new solutions $x_{i}^{t}$ and velocities $v_{i}^{t}$ at time step *t* are given by:

(3) $$\begin{array}{cc} \; & f_{i}=f_{min}+\left(f_{max}-f_{min}\right)\beta \\ \; & v_{i}^{t}=v_{i}^{t-1}+\left(x_{i}^{t}-x_{best}\right)f_{i}\\ \; & x_{i}^{t}=x_{i}^{t-1}+v_{i}^{t} \end{array}$$

where β∈[0,1] is a random vector drawn from a uniform distribution. Here, $x_{cgBest}$ is the current global best location (solution) which is located after comparing all the solutions among all the *n* bats.

For the local search part, once a solution is selected among the current best solutions, a new solution for each bat is generated locally using a random walk:

(4) $$\begin{array}{cc} \; & x_{new}=x_{old}+\epsilon A_{t} \end{array}$$

where ϵ∈[−1,1] is a random number, while $A_{t}=\langle A_{i}^{t}\rangle$ is the average loudness of all the bats at this time step.

Furthermore, the loudness $A_{i}$ and the rate $r_{i}$ of pulse emission have to be updated accordingly as the iterations proceed. These formulas are:

(5) $$\begin{array}{cc} \; & A_{i}^{t+1}=\alpha A_{i}^{t} \end{array}$$

(6) $$\begin{array}{cc} \; & r_{i}^{t+1}=r_{i}^{0}\left[1-exp\left(-\gamma t\right)\right] \end{array}$$

where α and γ are constants.

Based on these approximations and idealization, the pseudo-code of BA is shown in Algorithm 2.
**Algorithm 2:** Pseudocode for Bat Algorithm.
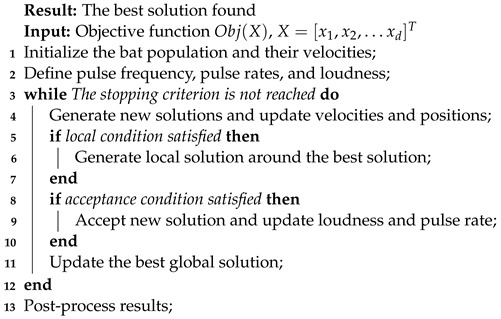


#### 3.1.3. Particle Swarm Optimization

PSO is a stochastic optimization method inspired by bird flocks and insect swarms. It has diverse applications including neural network training, function optimization, fuzzy control, and pattern classification [50,51]. Operates as follows:

1. Initialization: PSO begins with a population of particles in a search space. Each particle has an initial position and velocity.
2. Update: In each iteration, each particle adjusts its velocity and position based on rules derived from its personal experience and the global experience of the group.
3. Personal Experience: Each particle maintains a record of its best local position. Velocity $V_{i}\left(t\right)$ is the velocity of particle *i* at iteration *t* and position updates aim to converge towards this best local position $X_{i}\left(t\right)$ is the position of particle *i* at iteration *t*. Those formulas are described as follows:
(7) $$v_{i}\left(t+1\right)=w\cdot v_{i}\left(t\right)+c_{1}\cdot r_{1}\cdot \left(p_{i}\left(t\right)-x_{i}\left(t\right)\right)+c_{2}\cdot r_{2}\cdot \left(g\left(t\right)-x_{i}\left(t\right)\right)$$
(8) $$x_{i}\left(t+1\right)=x_{i}\left(t\right)+v_{i}\left(t+1\right)$$
4. Global Experience: The group of particles maintains a record of the best global position found. Particles also adjust their velocity and position to converge towards this best global position.
5. Stopping Criterion: The algorithm continues to iterate until a stopping criterion is satisfied, such as reaching a maximum number of iterations.

In our particle swarm optimization model, each particle’s movement is defined by several key elements: $v_{i}\left(t\right)$ represents the velocity of particle *i* at iteration *t*, determining its speed and direction. $x_{i}\left(t\right)$ indicates the position of particle *i* at iteration *t*, showing its location in the search space. $p_{i}\left(t\right)$ is the best position personally found by particle *i* up to iteration *t*, while g(t) denotes the best global position found by any particle up to iteration *t*, representing the best overall solution so far. the inertia weight *w* affects the particle’s momentum and its changes in direction. the acceleration coefficients $c_{1}$ and $c_{2}$ determine the particle’s movement towards its personal best and the global best positions, respectively. Lastly, $r_{1}$ and $r_{2}$ are random numbers between 0 and 1 that add an element of randomness to the particle’s path.

To delineate the behavior of this metaheuristic, we present its pseudocode in Algorithm 3.
**Algorithm 3:** Pseudocode for Particle Swarm Optimization.

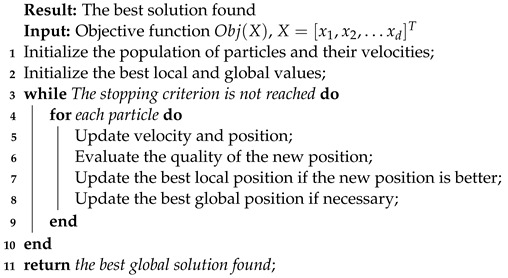



### 3.2. SWEVOH Logic

The goal of this component is to dynamically vary the population of the MH with characteristics that enable it to enhance its search space. This involves intensifying by minimizing the population and diversifying by increasing it.

Expanding on this idea, the population variation is a crucial aspect in optimizing the metaheuristic’s performance. When minimizing the population, the algorithm focuses on intensifying its search, aiming for a more concentrated exploration of promising regions. On the other hand, increasing the population facilitates diversification, allowing the algorithm to explore a broader solution space and potentially discover novel, optimal solutions. This dynamic adjustment of the population size plays a pivotal role in balancing between exploitation and exploration, contributing to the adaptability and effectiveness of the metaheuristic across different problem landscapes.

The SWEVOH component’s operational aspects are clarified by using a standard swarm method as an illustrative case to explain its functioning, parameter setup, and execution logic. the integrated SWEVOH Algorithm takes on the responsibility of dynamically supervising and adapting the bat population in response to solution performance and observed enhancements. Its pivotal role involves the continuous adjustment of the algorithm’s population size and composition throughout the optimization procedure. We incorporate the SWEVOH component at the outset of the iterative cycle for each metaheuristic. This ensures that, when the freedom parameter permits population adjustments, the metaheuristic’s continuity proceeds along its regular course. This is exemplified in Algorithm 4, specifically between lines 5 and 7, where it evaluates the need for the self-adaptive strategy in each iteration. Algorithm 4 is intended solely to illustrate the integration point of the autonomous component within each metaheuristic, positioned at the onset of iterations. This autonomous component dynamically adjusts the population size, aligning with the intensification and diversification criteria inherent to each metaheuristic.   
**Algorithm 4:** Standard Swarm pseudo code with SWEVOH component.

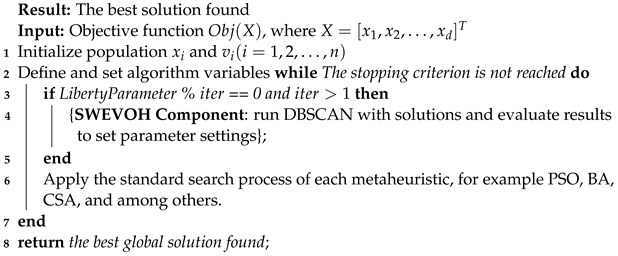



We introduce a flexibility parameter to regulate when to intervene with metaheuristic parameter values. This ensures the metaheuristic maintains its distinctive behavior. After one hundred unrestricted iterations, the algorithm checks for updates based on improvement percentages compared to predefined thresholds.

(9) $$\mathrm{improvement}\text{\_}\mathrm{percentage}=\frac{\mathrm{past}\text{\_}\mathrm{best}-\mathrm{current}\text{\_}\mathrm{best}}{\mathrm{past}\text{\_}\mathrm{best}}\times 100$$

- If the improvement percentage exceeds the defined acceptance value, the component will take the solutions with the worst results and remove them from the search space according to the logic explained in Section 3.3.
- If the improvement percentage falls below the accepted threshold, indicating insufficient progress, self-tuning strategies adjust the population.

### 3.3. Self-Tuning Strategies

The following self-tuning strategies are applied when the improvement percentage is below the accepted threshold:

1. Calculation of Clusters: DBSCAN partitions the population into clusters denoted as $C=\{C_{1},C_{2},\cdots ,C_{k}\}$ . For the separation of solutions, DBSCAN considers each solution vector $X_{n}$ as an element for processing, where *n* is the current population size. To calculate the distance between solutions, DBSCAN performs element-wise distance calculations hamming distance. Once DBSCAN performs the clustering of solutions, the average of each obtained cluster is calculated. The average fitness of each cluster $AVGC_{i}$ is computed as:
$$AVGC_{i}=\frac{1}{|C_{i}|}\sum_{x\in C_{i}}\mathrm{Fitness}\left(x\right)$$
  Here, $|C_{i}|$ denotes the number of solutions in cluster $C_{i}$ and Fitness(x) represents the evaluation of the solution *x* in the objective function. Each cluster is assessed based on the average fitness it contains to determine the sector where population size adjustments should be applied. The least-performing solutions from the worst fitness cluster ( $C_{\mathrm{worst}}$ ) will be employed to reduce the population size, while the best solutions ( $C_{\mathrm{best}}$ ) from the best fitness cluster will be utilized to increase the population size.
  The use of fitness-based cluster evaluations guides precise population modifications, ensuring a balanced and effective population management strategy.
2. Population Variation: In the process of expanding the population within defined constraints, a meticulous verification ensures that the addition of new solutions aligns with the set maximum population size $N_{\mathrm{Population}\text{\_}\max}$ . Selecting Increment_solutions_x_cluster superior solutions from the cluster boasting the highest fitness ( $C_{\mathrm{best}}$ ) initiates the generation phase. The quantity of newly generated solutions is regulated by the parameter Increment_solutions_x_cluster , maintaining a controlled expansion. Crucially, the logic governing the creation of these solutions remains faithful to the core principles intrinsic to each respective metaheuristic. In the scenario where it is feasible to reduce the population, ensuring it does not fall below the specified parameter $N_{\mathrm{Population}\text{\_}\min}$ , a procedure analogous to the one described earlier is implemented. This involves considering the Increment_solutions_x_cluster parameter negatively, applied to the cluster with the worst fitness performance ( $C_{\mathrm{worst}}$ ).
3. Replacement of solution: If the calculated clusters exhibit an average of solutions within an acceptable parameter range ( Diff_cluster_%_accepted ), it suggests that all solutions are converging to a local stagnation point. To address this, we replace half of the solutions in each underperforming cluster with new random solutions generated using the functions specific to each metaheuristic.

The SWEVOH component dynamically manages the metaheuristic population by monitoring the algorithm’s performance evolution. The population is kept stable if significant enhancements are noted. However, if the improvements are deemed insufficient, the function deploys self-tuning strategies to alter the population’s size and composition, all the while considering the clustering structure of the solutions. This adaptive method enables the algorithm to effectively navigate the search space and adjust to the continuously changing optimization landscapes.

Finally, it is crucial reinforcing that the computational complexity of the metaheuristics employed in this study is generally O(kn) , where *n* represents the dimension of the problem and *k* stands for the constant combining the total number of iterations or generations with the population size. This denotes the cumulative number of objective function evaluations conducted throughout the execution of the algorithm. Additionally, at specific intervals, the complexity of the DBSCAN algorithm, typically O(nlogn) , must be considered. This is due to the potential necessity for comparing all pairs of points within the dataset. Incorporating DBSCAN’s complexity into the metaheuristics framework undeniably elevates the overall computational cost. However, this increase is justifiable given the enhanced outcomes achieved. Furthermore, it is important to note the ever-increasing accessibility of computing power, driven by continuous technological advancements, which helps mitigate the impact of this added complexity.

## 4. Experimental Results

To evaluate the performance of our proposal, we test the CEC LSGO functions, comparing the original metaheuristics with standard configurations, versus the SWEVOH.

### 4.1. Methodology

**Figure 1:**
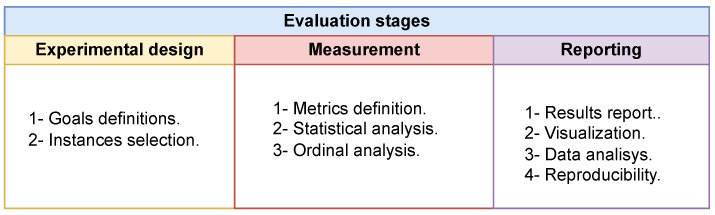
Evaluation stages to determine the performance of an metaheuristic.

To adequately evaluate the performance of metaheuristics, a performance analysis is required [52]. For this work, we compare the supplied best solution of the SWEVOH to the best-known result of the benchmark retrieved from [17]. Figure 1 depicts the procedures involved in doing a thorough examination of the enhanced metaheuristic. We create objectives and recommendations for the experimental design to show that the proposed approach is a viable alternative for determining metaheuristic parameters. Then, as a vital indicator for assessing future results, we evaluate the best value. We use ordinal analysis and statistical testing to evaluate whether a strategy is significantly better in this circumstance. Lastly, we detail the hardware and software aspects that were used to replicate computational experiments, and we present all of the results in tables and graphs.

**Figure 2:**
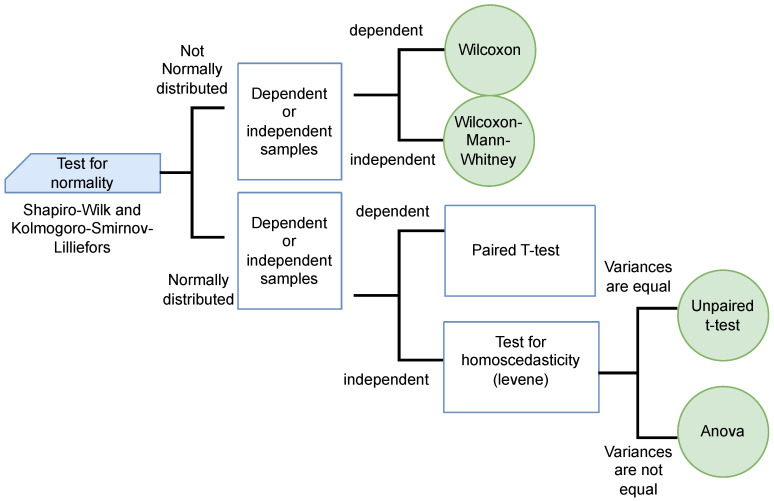
Statistical significance test.

As a result, we conduct a contrast statistical test for each case, using the Kolmogorov–Smirnov–Lilliefors process [53] to measure sample autonomy and the Mann–Whitney–Wilcoxon [54] test to statistically evaluate the data. In Figure 2, we describe and determine the organization.

The Kolmogorov–Smirnov–Lilliefors test allows us to assess sample independence by calculating the $Z_{MIN}$ or $Z_{MAX}$ (depending on whether the task is minimization or maximization) obtained from each instance’s 31 executions.

All functions within the CEC LSGO reach their maximum efficiency at 0; hence, in order to discern the most favorable values obtained, it is essential to ascertain those that approach zero or are in close proximity to this value. The best obtained values can be visualized in bold.

### 4.2. CEC LSGO Function Results

Infrastructure: Python 3.10 was used to implement SWEVOH. The computer has the common attributes: MacOS with a 2.7 GHz Intel Core i7 CPU and 16 GB of RAM.

Setup variables: The configuration for our suggested approach is shown in Table 1, Table 2, Table 3 and Table 4.

Fifteen functions were considered, each of which was run 31 times; the results are presented in Table 5, Table 6 and Table 7.

The algorithms are ranked in order of $Z_{min}$ achieved. The instances that obtained $Z_{min}$ are also displayed. As can be seen in the results of the algorithms that solved CEC LSGO function, we compare the distribution of the samples of each instance using a violin plot, which allows us to observe the entire distribution of the data. We provide and discuss the most difficult instances of each group to create a resume of all the instances below:

The information is organized as follows: MIN: the minimum value reached; MAX: the maximum value reached; MEAN: the average value.

The first method was the standard cuckoo search algorithm with various settings, and the second was SACSDBSCAN, as previously indicated.

As depicted in Table 5, the comparative analysis showcases the performance disparities between the Original PSO Algorithm and the Autonomous PSO Algorithm across various high-density functions from the CEC. The table delineates the minimum, maximum, and mean values for both algorithmic versions concerning these functions. Notably, upon examination, a trend emerges wherein the Autonomous PSO Algorithm demonstrates superior performance in terms of minimum values across the majority of functions. This trend suggests an enhanced search capability exhibited by the Autonomous PSO Algorithm compared to the Original PSO, signifying its efficacy in exploring the solution space to discover more optimal or near-optimal solutions, particularly evident in its consistently lower minimum values across diverse functions.

**Figure 3:**
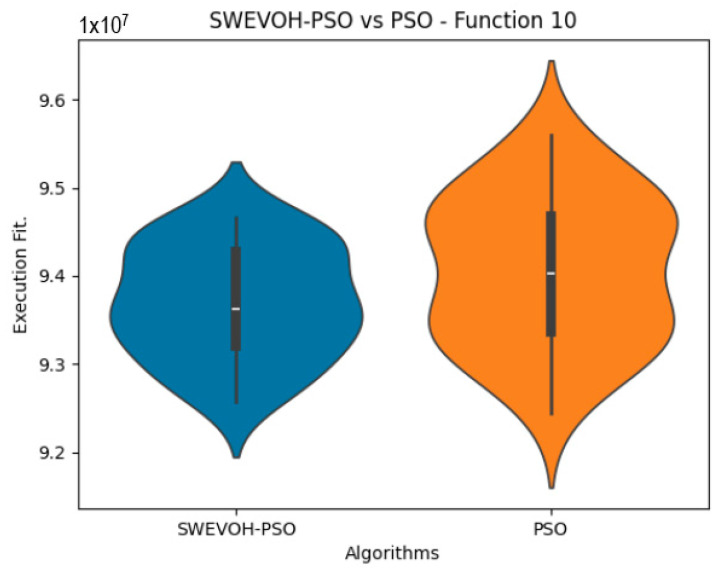
SWEVO-PSO vs. PSO distribution on F10.

**Figure 4:**
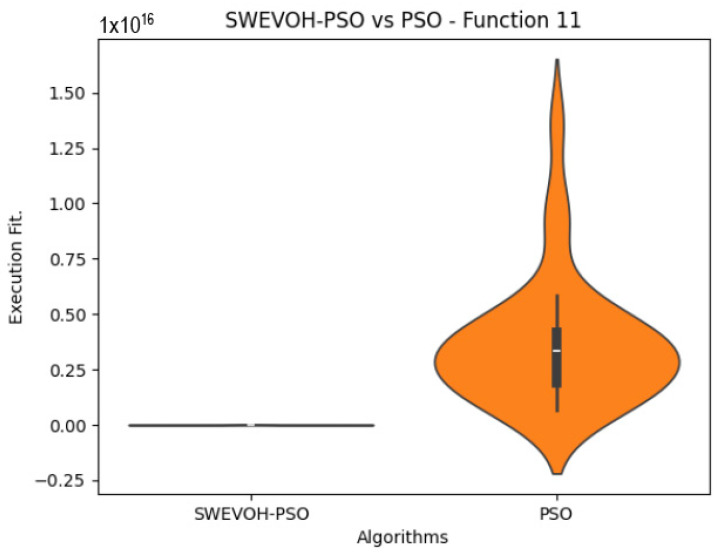
SWEVO-PSO vs. PSO distribution on F11.

**Figure 5:**
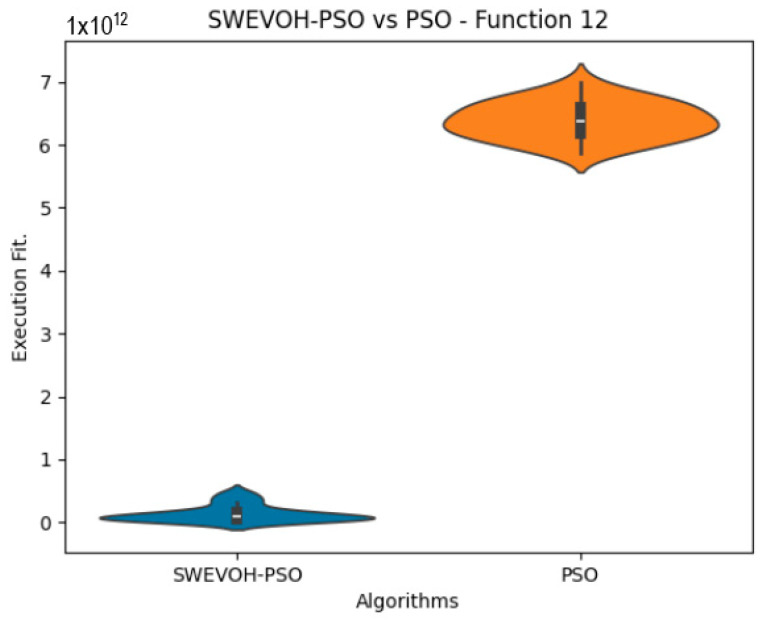
SWEVO-PSO vs. PSO distribution on F12.

**Figure 6:**
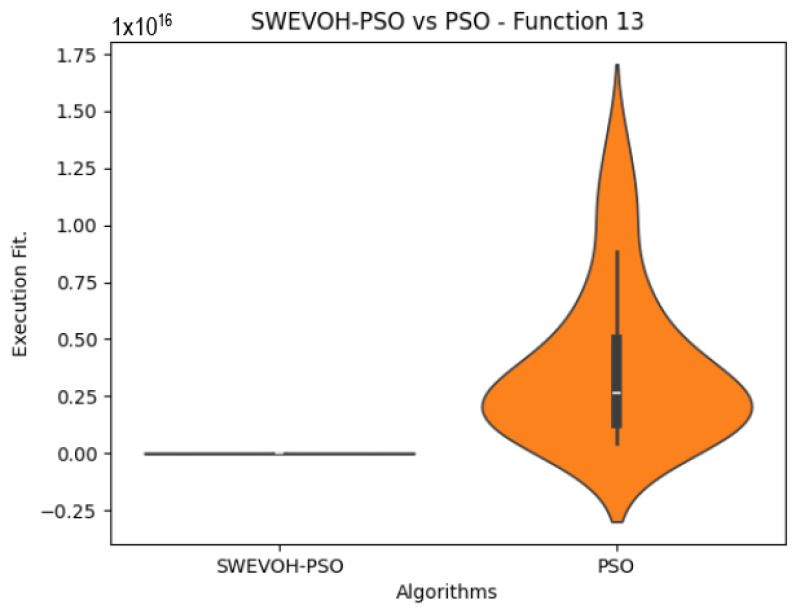
SWEVO-PSO vs. PSO distribution on F13.

**Figure 7:**
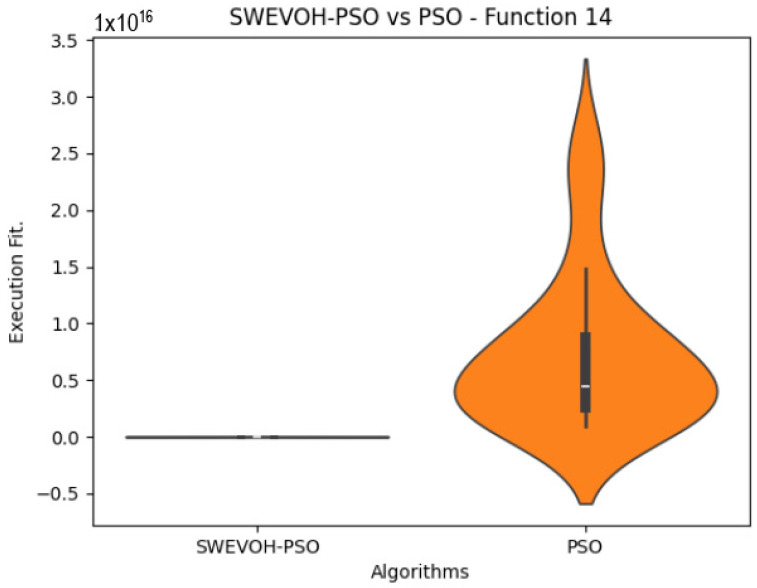
SWEVO-PSO vs. PSO distribution on F14.

**Figure 8:**
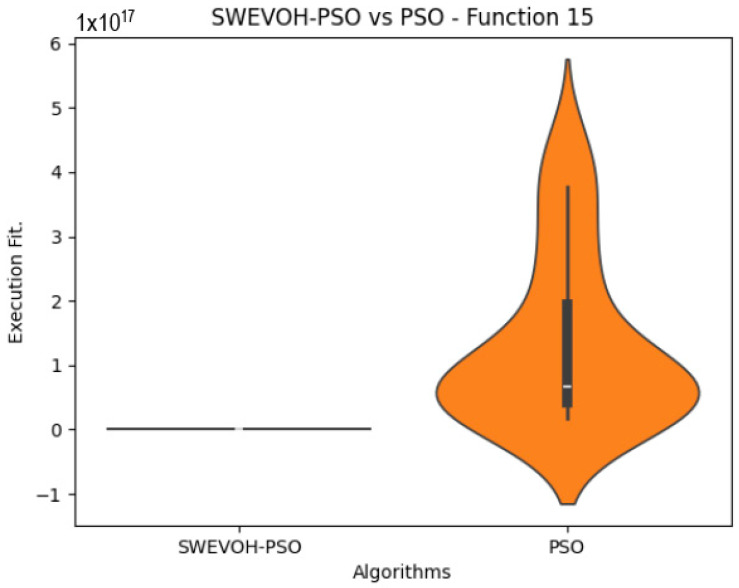
SWEVO-PSO vs. PSO distribution on F15.

In the distribution of the data in the functions 10 to 15 (Figure 3, Figure 4, Figure 5, Figure 6, Figure 7 and Figure 8), as we can observe the behavior of our proposal aligns with the hybrid logic as initially envisaged. This allows for a broader search space coverage, concentrating the highest density of optimal values near the vicinity of the known minimum.

Table 6 offers a comprehensive comparison between two variants of the Cuckoo Search Algorithm (CSA): the Original Cuckoo Search Algorithm and the Autonomous Cuckoo Search Algorithm across multiple high-density functions derived from the CEC dataset. The table presents essential statistical values including minimum, maximum, and mean values obtained for each function under both algorithmic versions.

Notably, upon analysis, it becomes evident that the Autonomous Cuckoo Search Algorithm consistently outperforms the Original Cuckoo Search Algorithm, particularly in achieving superior minimum values across a majority of the evaluated functions. This observed trend signifies a noteworthy improvement in the efficiency and efficacy of the Autonomous Cuckoo Search Algorithm compared to its original counterpart. The ability of the Autonomous variant to consistently yield better minimum values suggests a heightened capability to explore and discover more optimal or near-optimal solutions within the solution space for a diverse set of functions, showcasing its enhanced algorithmic effectiveness and potential for improved performance in optimization tasks.

**Figure 9:**
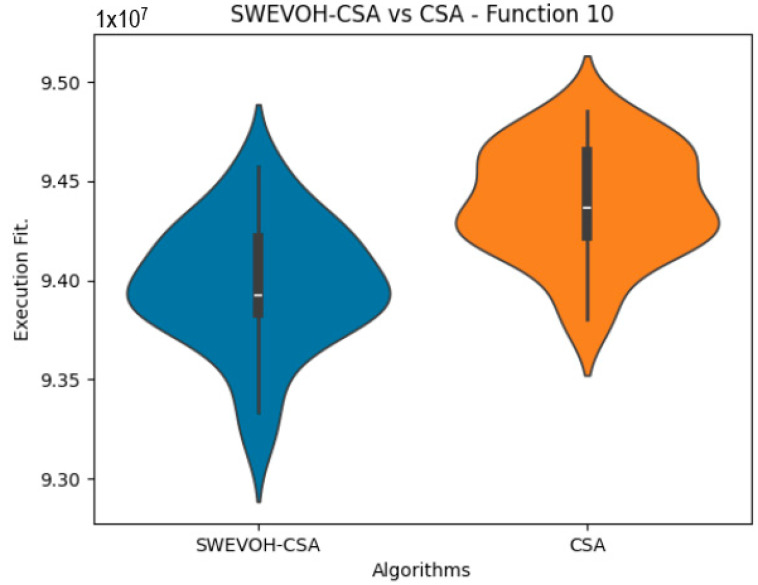
SWEVO-CSA vs. CSA distribution on F10.

**Figure 10:**
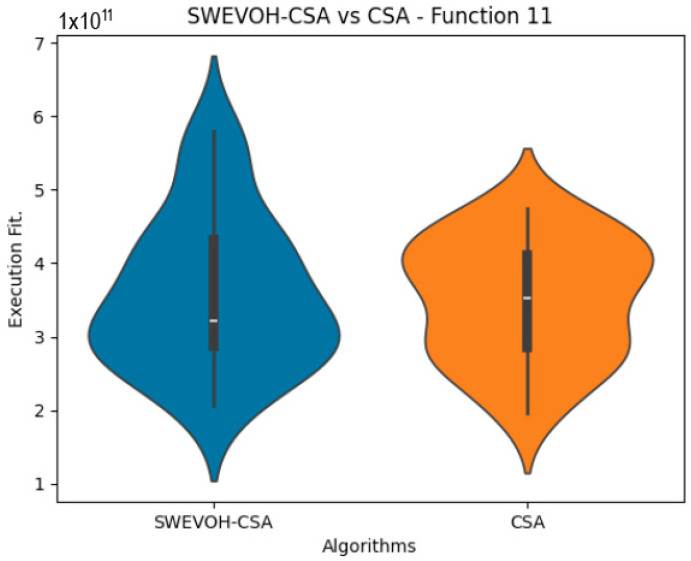
SWEVO-CSA vs. CSA distribution on F11.

**Figure 11:**
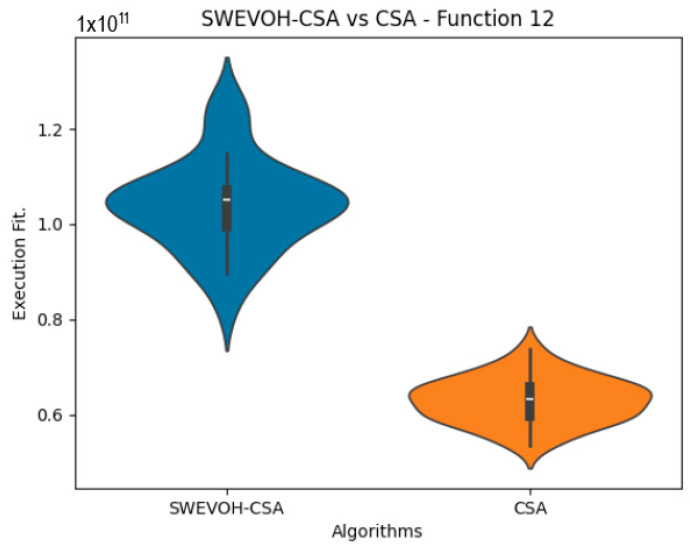
SWEVO-CSA vs. CSA distribution on F12.

**Figure 12:**
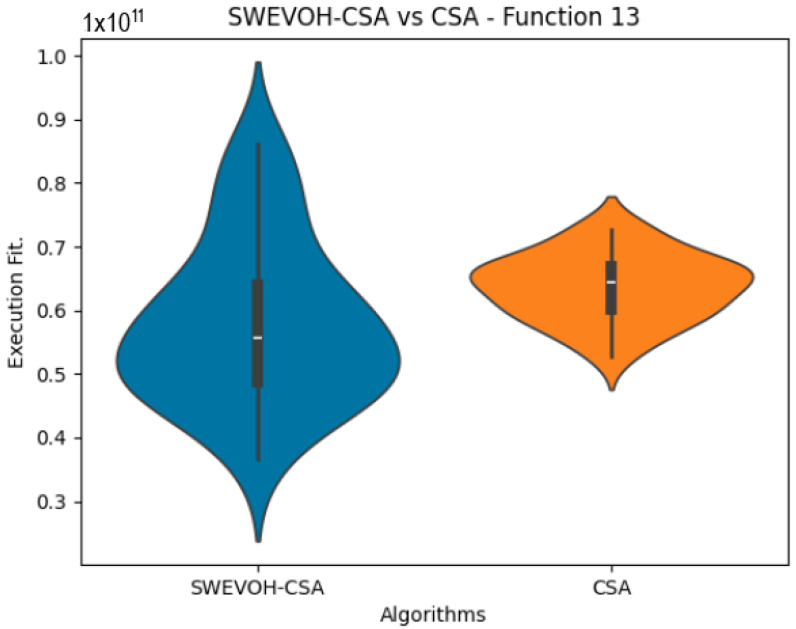
SWEVO-CSA vs. CSA distribution on F13.

**Figure 13:**
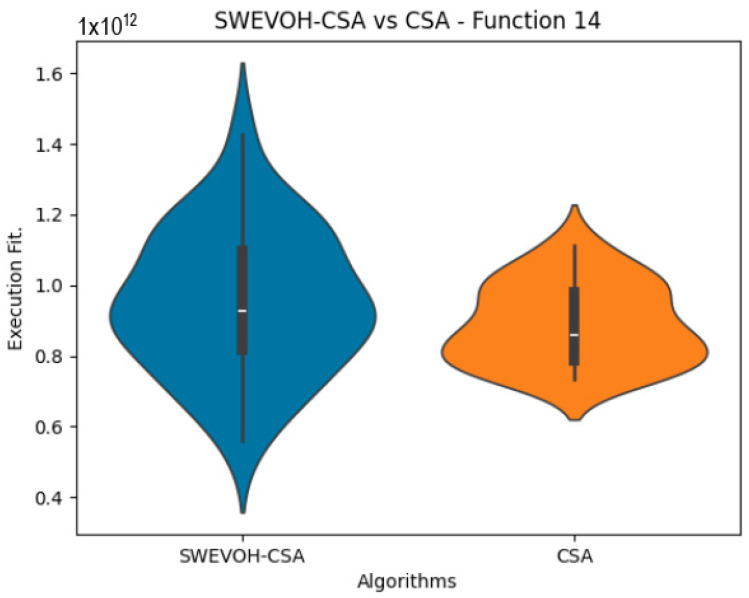
SWEVO-CSA vs. CSA distribution on F14.

**Figure 14:**
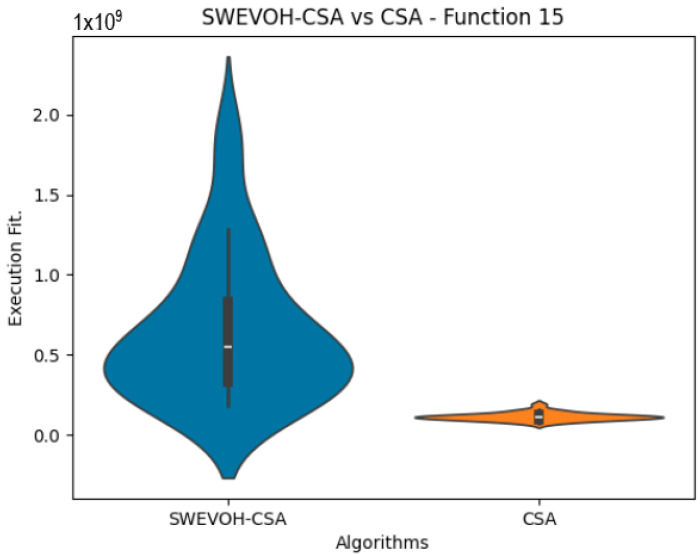
SWEVO-CSA vs. CSA distribution on F15.

Similar to the images observed in the PSO algorithm, the distribution of our proposal remains consistent in its shape and behavior. The images (Figure 9, Figure 10, Figure 11, Figure 12, Figure 13 and Figure 14) demonstrate that for the indicated functions, the distribution of the 31 executions enables a more effective exploration of the search space, facilitating the discovery of solutions that lead to improved optimal values.

Table 7 presents a detailed comparison between two variations of the Bat Algorithm (BA): the Original Bat Algorithm and the Autonomous Bat Algorithm, denoted as SWEVOH-BA. This comparison encompasses a range of high-density functions from the CEC dataset, illustrating essential statistical values including minimum, maximum, and mean values for each function under both algorithmic versions.

Upon analysis, a notable trend emerges: the Autonomous Bat Algorithm consistently demonstrates notably superior minimum values when compared to the Original Bat Algorithm across various functions. This significant difference in minimum values implies a tangible enhancement in the performance and optimization capabilities of the Autonomous Bat Algorithm. Specifically, the consistently lower minimum values achieved by the Autonomous variant suggest its heightened efficiency in exploring the solution space and locating more optimal or near-optimal solutions across diverse functions.

This observed improvement in achieving better minimum values signifies the efficacy and potential superiority of the Autonomous Bat Algorithm in optimizing and solving optimization problems, showcasing its enhanced performance compared to the Original Bat Algorithm.

**Figure 15:**
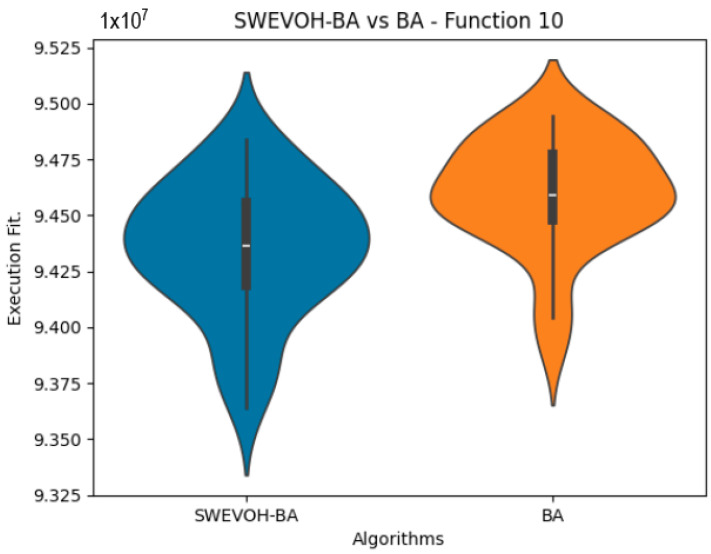
SWEVO-BA vs. BA distribution on F10.

**Figure 16:**
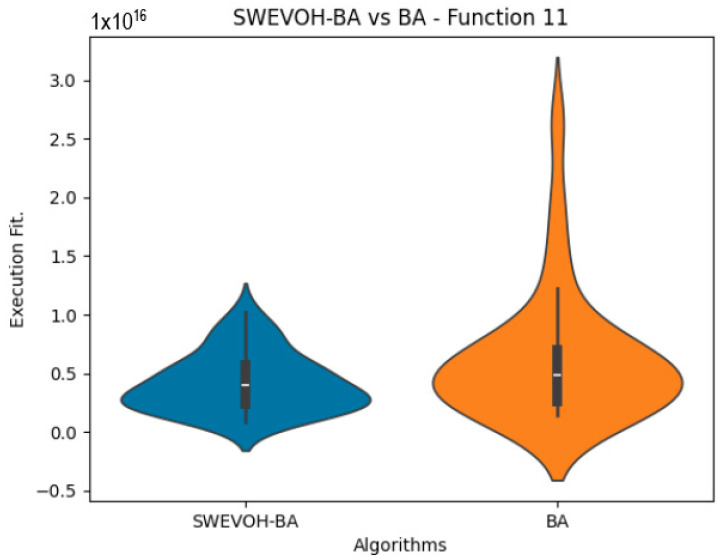
SWEVO-BA vs. BA distribution on F11.

**Figure 17:**
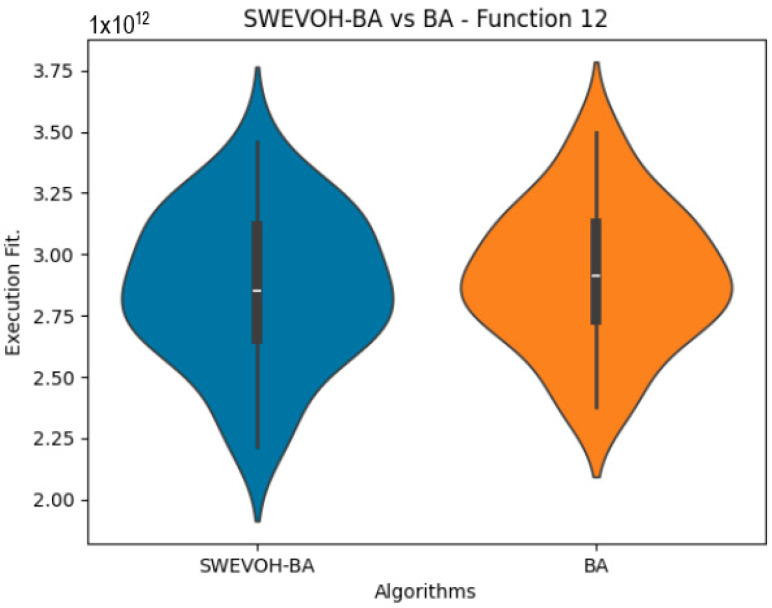
SWEVO-BA vs. BA distribution on F12.

**Figure 18:**
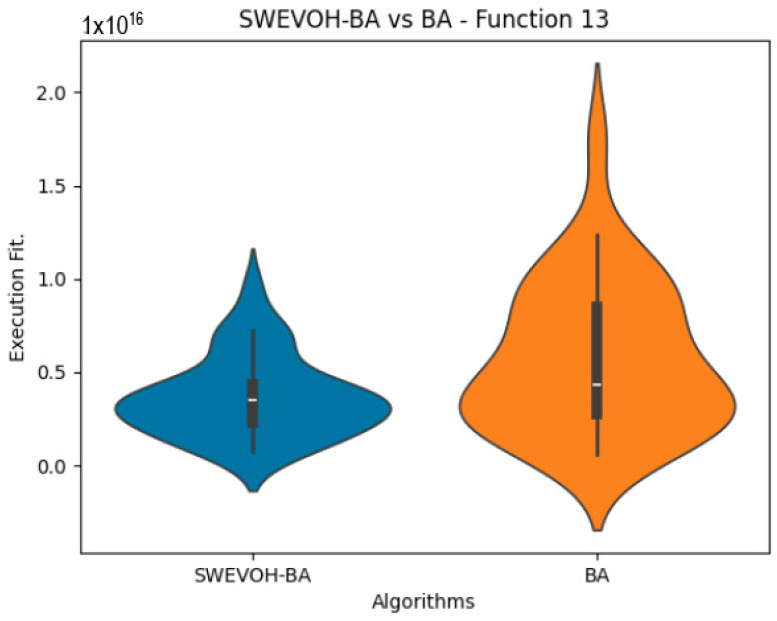
SWEVO-BA vs. BA distribution on F13.

**Figure 19:**
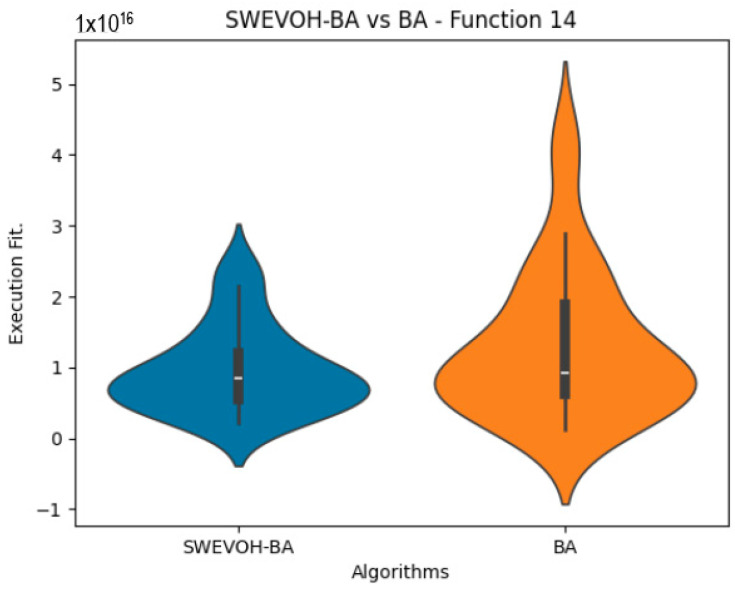
SWEVO-BA vs. BA distribution on F14.

**Figure 20:**
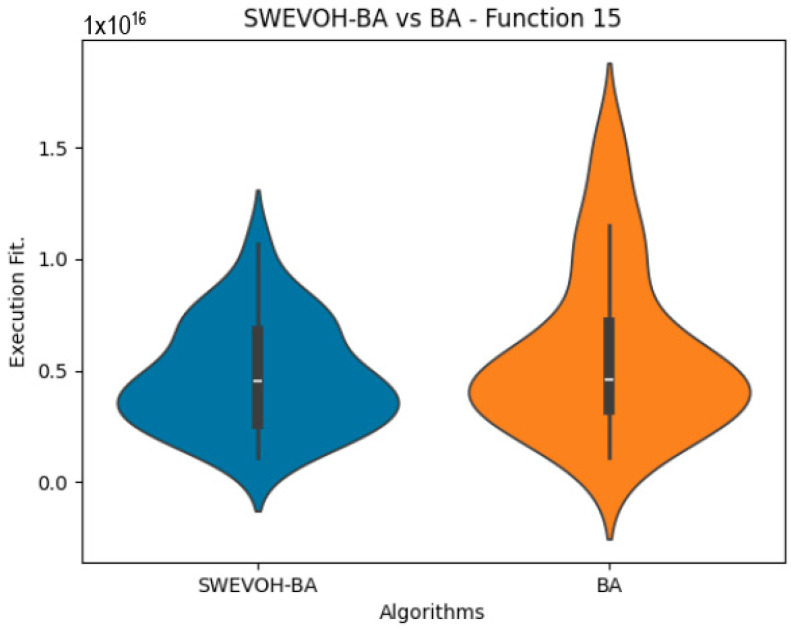
SWEVO-BA vs. BA distribution on F15.

Similar to the images observed in the BA algorithm, the distribution of our proposal remains consistent in its shape and behavior. Images (Figure 15, Figure 16, Figure 17, Figure 18, Figure 19 and Figure 20), demonstrate that for the indicated functions, the distribution of the 31 executions enables a more effective exploration of the search space, facilitating the discovery of solutions that lead to improved optimal values.

**Figure 21:**
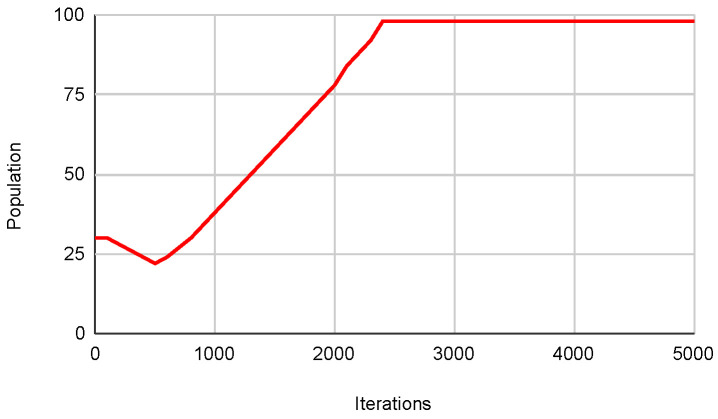
Pop. in SWEVOH-BA—Func. 2.

**Figure 22:**
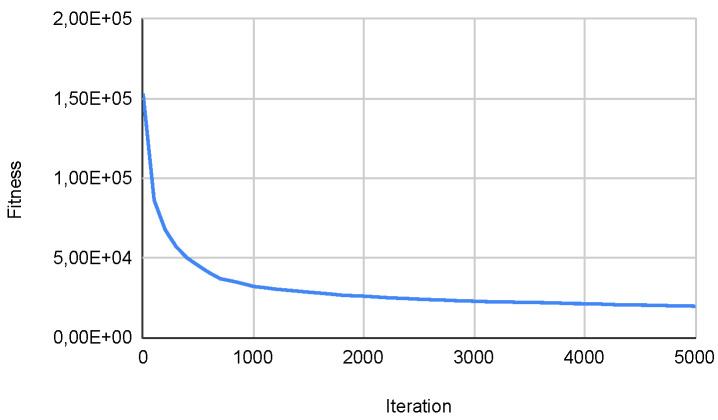
Convg. in SWEVOH-BA—Func. 2.

**Figure 23:**
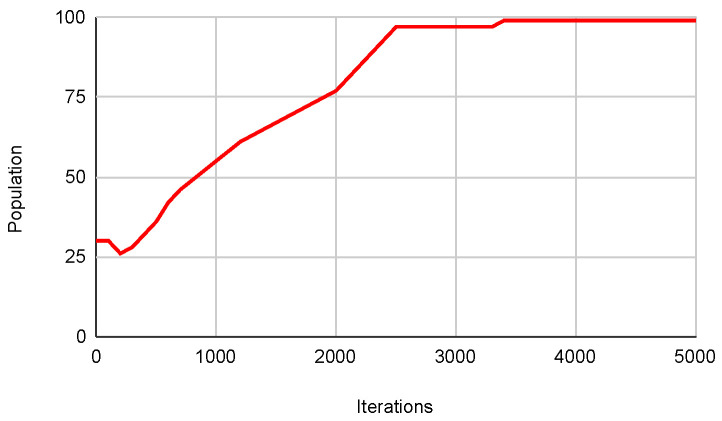
Pop. in SWEVOH-CSA—Func. 9.

**Figure 24:**
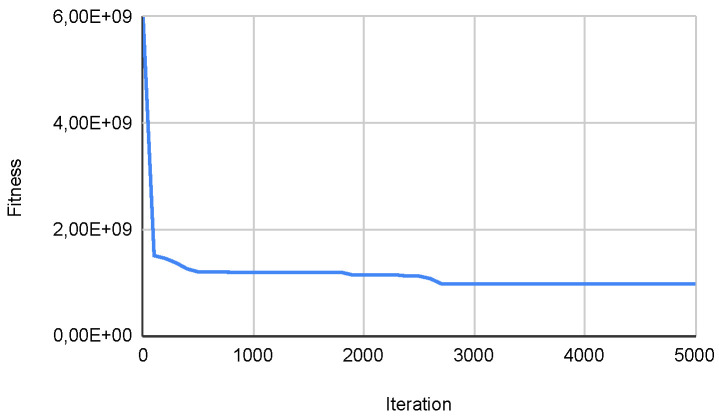
Convg. in SWEVOH-CSA—Func. 9.

Overall, the results indicate that autonomous algorithms enhance performance compared to their original counterparts in most high-density functions from the CEC. Specifically, the Autonomous PSO Algorithm achieves better minimum results in most functions. The Autonomous Cuckoo Search Algorithm also outperforms the Original Cuckoo Search in terms of minimum values in multiple functions. Lastly, the Autonomous Bat Algorithm demonstrates superior performance compared to the Original Bat Algorithm in terms of minimum values in several functions. Furthermore, Figure 21 below shows how the population dynamically adjusts according to what is described in the Section 3.3, which is directly related to what is observed in Figure 22 that shows how the metaheuristic works with an expected convergence, which has caused the number of the population to increase. Another example of what was described above can be seen in the Figure 23 and Figure 24 that belongs to function 9 in Table 8.

### 4.3. Statistical Test

We present the following hypotheses to assess independence.

-
$H_{0}$ : states that $Z_{min}$ / $Z_{max}$ follows a normal distribution.
-
$H_{1}$ : states the opposite.

The test resulted in a *p*-value below 0.05, indicating that $H_{0}$ cannot be assumed. With independent samples and a non-normal distribution, the central limit theorem does not apply. Therefore, we employ the non-parametric Mann–Whitney–Wilcoxon test to assess heterogeneity in the results of the most challenging instances. We propose the following hypotheses:

-
$H_{0}$ : CSA is better than SACSDBSCAN
-
$H_{1}$ : states the opposite.

The statistical contrast test ultimately determines which technique is significantly superior.

The Wilcoxon signed rank test was used to compare LSGO CEC function on the algorithms techniques. Smaller values than 0.05 define that $H_{0}$ cannot be assumed because the significance level is also set to 0.05 .

To conduct the test run that supports the study, we use a method from the PISA system. We specify all data distributions (each in a file and each data in a line) in this procedure, and the algorithm returns a *p*-value for the hypotheses.

The following tables show the result of the Mann–Whitney–Wilcoxon test. To understand them, it is necessary to know the following acronyms:

- SWS = Statistically without significance.

SWEVOH-PSO vs. PSO—*p*-value

The *p*-values for each function’s results are presented in Table 9, Table 10, Table 11, Table 12, Table 13, Table 14, Table 15, Table 16, Table 17, Table 18, Table 19, Table 20, Table 21, Table 22 and Table 23. In 8 of 15 cases, the reported *p*-values are less than 0.05, indicating a statistically significant difference between SWEVOH-PSO and PSO for evaluated functions. Hence, we can conclude that SWEVOH-PSO is statistically superior to PSO in those evaluated functions in this study.

SWEVOH-CSA vs. CSA—*p*-value.

The *p*-values for each function’s results are presented in Table 24, Table 25, Table 26, Table 27, Table 28, Table 29, Table 30, Table 31, Table 32, Table 33, Table 34, Table 35, Table 36, Table 37 and Table 38. In 7 of 15 cases, as mentioned above, t. he *p*-values reported are less than 0.05, and SWS suggests that they have no statistical significance. So, with this knowledge, in each instance mentioned, we can see the SWEVOH-CSA algorithm was better than the original CSA. In four out of the remaining eight cases, none can demonstrate significant superiority over the other.

If we focus on the instances where our proposal improves the result obtained in comparison to the original CSA, we can infer that the solutions achieved are distributed in a centered way on their optimal value, which reflects that the behavior of this algorithm is according to the SWEVOH behavior. This is reflected in the violin graphs in Figure 9, Figure 10 and Figure 12.

SWEVOH-BA vs. BA—*p*-value.

The *p*-values for each function’s results are presented in Table 39, Table 40, Table 41, Table 42, Table 43, Table 44, Table 45, Table 46, Table 47, Table 48, Table 49, Table 50, Table 51, Table 52 and Table 53. In 8 of 15 scenarios, the *p*-values reported are less than 0.05, and 7 of the remaining cases are both SWS, which suggests that they have no statistical significance. So, with this knowledge, in each instance mentioned, we can see the SWEVOH-BA algorithm was better than the original BA.

## 5. Conclusions

In this study, we have proposed an integrated approach that combines metaheuristics and clustering techniques to address the challenge of dynamically managing solution populations in optimization algorithms. Our approach is based on three optimization metaheuristics: Particle Swarm Optimization (PSO), Cuckoo Search Algorithm (CSA), and Bat Algorithm (BA), which have consistently demonstrated good performance across a variety of optimization challenges.

Moreover, the DBSCAN clustering algorithm has been integrated to enhance the capability for dynamic management and categorization of solution populations. This incorporation facilitates the adjustment of solution numbers within the metaheuristic dynamically, increasing solutions when diversification is needed or reducing them during intensification scenarios.

The evaluation of our approach was conducted using the complex functions from the widely recognized CEC LSGO test suite in the optimization community. Our results demonstrate that our integrated approach achieves significantly better performance compared to traditional approaches of static population management. This is attributed to our approach’s capacity to dynamically adapt to changes in the optimization landscape and explore promising regions within the solution space.

Furthermore, we performed a comprehensive statistical analysis to assess the significance of the obtained results. We employed Kolmogorov–Smirnov–Lilliefors and Mann–Whitney–Wilcoxon tests to evaluate sample independence and conduct statistical comparisons, respectively. These analyses allowed us to conclusively demonstrate the superiority of our integrated approach in terms of performance.

It is essential to note that the replicability of our experiments was ensured by using standard hardware and software widely accepted in the optimization community. All obtained results are presented clearly and concisely in tables and graphs to facilitate their comprehension and analysis.

In summary, our integrated approach of metaheuristics and clustering proves to be a viable and effective alternative for the dynamic management of solution populations in optimization algorithms. Our results, supported by a comprehensive statistical analysis, substantiate that our approach significantly outperforms traditional approaches to static population management. This is attributed to our approach’s ability to dynamically adapt to changes in the optimization landscape and explore promising regions within the solution space.

Additionally, it is noteworthy that our experiments were replicable using standard hardware and software within the optimization community. All results obtained are presented clearly and concisely in tables and graphs, making them easily understandable and analyzable.

As part of our future endeavors, we aim to explore approaches to enhance the precision of solution narrowing, preserving the metaheuristic’s proficiency in discovering superior solutions.

In conclusion, our integrated approach offers an effective and promising solution to address the challenge of dynamically managing solution populations in optimization algorithms. The results obtained provide evidence for the superiority of our approach and lay the foundation for future research in this field.

## Figures and Tables

**Table 1:** SWEVOH Parameters for self-tunning population size.

Population Min–Max	Improve % Accepted	Increment Solutions × Cluster	Diff Cluster % Accepted
10–100	10%	2	5%

**Table 2:** SWEVOH CSA Parameters for CEC LSGO.

Population Initial	Abandon Probability $P_{a}$	α	Max Iterations	$L_{b}$ and $U_{b}$
30	0.25	0.01	5000	Acc. to each func.

**Table 3:** SWEVOH BA Parameters for CEC LSGO.

Population Initial	Loudness *A*	Pulse Rate *r*	α	γ	Max Iterations	$L_{b}$ and $U_{b}$
30	0.95	0.1	0.9	0.5	5000	Acc. to each func.

**Table 4:** SWEVOH PSO Parameters for CEC LSGO.

Population Initial	Max Iterations	$L_{b}$ and $U_{b}$
30	5000	Acc. to each func.

**Table 5:** Comparison results between Original PSO Algorithm and Autonomous PSO Algorithm.

	Original PSO			Autonomous PSO		
Function	Min	Max	Mean	Min	Max	Mean
f1	1.69 × $10^{11}$	5.62 × $10^{11}$	2.33 × $10^{11}$	**6.63 × $10^{9}$ **	5.52 × $10^{11}$	7.00 × $10^{10}$
f2	8.51 × $10^{4}$	1.71 × $10^{5}$	9.65 × $10^{4}$	**4.25 × $10^{4}$ **	1.66 × $10^{5}$	6.05 × $10^{4}$
f3	2.15 × $10^{1}$	2.18 × $10^{1}$	2.15 × $10^{1}$	**2.13 × $10^{1}$ **	2.18 × $10^{1}$	2.14 × $10^{1}$
f4	1.94 × $10^{12}$	6.42 × $10^{14}$	8.42 × $10^{12}$	**4.10 × $10^{11}$ **	5.46 × $10^{14}$	5.68 × $10^{12}$
f5	3.03 × $10^{7}$	2.09 × $10^{8}$	4.37 × $10^{7}$	**2.49 × $10^{7}$ **	2.44 × $10^{8}$	4.08 × $10^{7}$
f6	1.05 × $10^{6}$	1.09 × $10^{6}$	1.06 × $10^{6}$	**1.04 × $10^{6}$ **	1.09 × $10^{6}$	1.05 × $10^{6}$
f7	3.74 × $10^{12}$	8.76 × $10^{20}$	1.12 × $10^{18}$	**3.12 × $10^{9}$ **	1.05 × $10^{21}$	9.51 × $10^{17}$
f8	2.51 × $10^{16}$	4.66 × $10^{19}$	3.87 × $10^{17}$	**1.10 × $10^{15}$ **	4.29 × $10^{19}$	2.60 × $10^{17}$
f9	2.34 × $10^{9}$	3.10 × $10^{10}$	3.57 × $10^{9}$	**2.06 × $10^{9}$ **	6.48 × $10^{10}$	3.44 × $10^{9}$
f10	9.24 × $10^{7}$	9.96 × $10^{7}$	9.41 × $10^{7}$	**9.09 × $10^{7}$ **	9.95 × $10^{7}$	9.39 × $10^{7}$
f11	6.55 × $10^{14}$	7.04 × $10^{23}$	8.60 × $10^{20}$	**6.83 × $10^{11}$ **	3.17 × $10^{23}$	4.19 × $10^{20}$
f12	5.86 × $10^{12}$	1.09 × $10^{13}$	6.46 × $10^{12}$	**1.75 × $10^{10}$ **	1.13 × $10^{13}$	1.46 × $10^{12}$
f13	4.30 × $10^{14}$	1.56 × $10^{23}$	2.80 × $10^{20}$	**2.00 × $10^{11}$ **	5.48 × $10^{23}$	3.84 × $10^{20}$
f14	8.90 × $10^{14}$	1.27 × $10^{23}$	9.74 × $10^{19}$	**2.00 × $10^{12}$ **	6.50 × $10^{23}$	4.17 × $10^{20}$
f15	1.64 × $10^{16}$	2.08 × $10^{19}$	3.04 × $10^{17}$	**2.61 × $10^{8}$ **	2.64 × $10^{19}$	2.07 × $10^{17}$

**Table 6:** Comparison results between Original Cuckoo Search Algorithm versus Autonomous Cuckoo Search Algorithm.

	Original CSA			Autonomous CSA		
Function	Min	Max	Mean	Min	Max	Mean
f1	**2.21 × $10^{8}$ **	5.88 × $10^{11}$	3.07 × $10^{10}$	3.65 × $10^{8}$	5.67 × $10^{11}$	3.03 × $10^{10}$
f2	2.02 × $10^{4}$	1.67 × $10^{5}$	3.18 × $10^{4}$	**1.97 × $10^{4}$ **	1.70 × $10^{5}$	3.16 × $10^{4}$
f3	2.15 × $10^{1}$	2.18 × $10^{1}$	2.15 × $10^{1}$	**2.14 × $10^{1}$ **	2.18 × $10^{1}$	2.15 × $10^{1}$
f4	3.48 × $10^{11}$	8.15 × $10^{14}$	6.91 × $10^{12}$	**2.60 × $10^{11}$ **	7.74 × $10^{14}$	6.53 × $10^{12}$
f5	1.45 × $10^{7}$	2.33 × $10^{8}$	2.23 × $10^{7}$	**9.63 × $10^{6}$ **	2.24 × $10^{8}$	1.82 × $10^{7}$
f6	1.06 × $10^{6}$	1.09 × $10^{6}$	1.07 × $10^{6}$	**1.05 × $10^{6}$ **	1.09 × $10^{6}$	1.06 × $10^{6}$
f7	2.09 × $10^{9}$	5.67 × $10^{20}$	1.35 × $10^{18}$	**1.82 × $10^{9}$ **	1.19 × $10^{21}$	1.13 × $10^{18}$
f8	3.04 × $10^{15}$	5.95 × $10^{19}$	2.96 × $10^{17}$	**1.87 × $10^{15}$ **	5.07 × $10^{19}$	2.94 × $10^{17}$
f9	1.17 × $10^{9}$	2.44 × $10^{11}$	2.07 × $10^{9}$	**7.44 × $10^{8}$ **	3.24 × $10^{10}$	1.58 × $10^{9}$
f10	9.38 × $10^{7}$	9.95 × $10^{7}$	9.47 × $10^{7}$	**9.29 × $10^{7}$ **	9.93 × $10^{7}$	9.44 × $10^{7}$
f11	**1.96 × $10^{11}$ **	6.58 × $10^{23}$	6.75 × $10^{20}$	2.07 × $10^{11}$	7.34 × $10^{23}$	7.77 × $10^{20}$
f12	**5.35 × $10^{10}$ **	1.08 × $10^{13}$	9.27 × $10^{11}$	7.76 × $10^{10}$	1.10 × $10^{13}$	9.42 × $10^{11}$
f13	5.28 × $10^{10}$	1.91 × $10^{23}$	1.73 × $10^{20}$	**3.66 × $10^{10}$ **	4.11 × $10^{22}$	3.34 × $10^{19}$
f14	7.34 × $10^{11}$	8.14 × $10^{24}$	5.18 × $10^{21}$	**5.59 × $10^{11}$ **	1.46 × $10^{23}$	1.63 × $10^{20}$
f15	**7.06 × $10^{7}$ **	2.13 × $10^{19}$	1.55 × $10^{17}$	1.85 × $10^{8}$	2.18 × $10^{19}$	1.49 × $10^{17}$

**Table 7:** Comparison results between Original BA versus SWEVOH -BA.

	Original BAT			Autonomous Bat		
Function	Min	Max	Mean	Min	Max	Mean
f1	1.71 × $10^{11}$	4.35 × $10^{11}$	2.04 × $10^{11}$	**1.59 × $10^{11}$ **	4.18 × $10^{11}$	2.01 × $10^{11}$
f2	2.86 × $10^{4}$	1.31 × $10^{5}$	3.55 × $10^{4}$	**2.55 × $10^{4}$ **	1.32 × $10^{5}$	3.45 × $10^{4}$
f3	2.16 × $10^{1}$	2.17 × $10^{1}$	2.16 × $10^{1}$	**2.16 × $10^{1}$ **	2.17 × $10^{1}$	2.16 × $10^{1}$
f4	**1.49 × $10^{12}$ **	8.17 × $10^{13}$	6.16 × $10^{12}$	2.16 × $10^{12}$	1.03 × $10^{14}$	5.33 × $10^{12}$
f5	1.15 × $10^{7}$	9.10 × $10^{7}$	1.65 × $10^{7}$	**1.12 × $10^{7}$ **	1.02 × $10^{8}$	1.67 × $10^{7}$
f6	**1.06 × $10^{6}$ **	1.08 × $10^{6}$	1.06 × $10^{6}$	1.06 × $10^{6}$	1.08 × $10^{6}$	1.06 × $10^{6}$
f7	1.49 × $10^{13}$	1.30 × $10^{17}$	6.06 × $10^{14}$	**7.55 × $10^{12}$ **	7.55 × $10^{16}$	5.19 × $10^{14}$
f8	1.96 × $10^{16}$	3.56 × $10^{18}$	2.33 × $10^{17}$	**1.74 × $10^{16}$ **	4.98 × $10^{18}$	2.71 × $10^{17}$
f9	1.02 × $10^{9}$	8.73 × $10^{9}$	1.43 × $10^{9}$	**9.79 × $10^{8}$ **	9.15 × $10^{9}$	1.36 × $10^{9}$
f10	9.39 × $10^{7}$	9.75 × $10^{7}$	9.48 × $10^{7}$	**9.36 × $10^{7}$ **	9.74 × $10^{7}$	9.47 × $10^{7}$
f11	1.41 × $10^{15}$	4.99 × $10^{18}$	3.94 × $10^{16}$	**8.98 × $10^{14}$ **	8.47 × $10^{18}$	4.30 × $10^{16}$
f12	2.38 × $10^{12}$	8.94 × $10^{12}$	3.03 × $10^{12}$	**2.22 × $10^{12}$ **	8.93 × $10^{12}$	3.00 × $10^{12}$
f13	**5.75 × $10^{14}$ **	7.02 × $10^{18}$	4.82 × $10^{16}$	7.85 × $10^{14}$	4.02 × $10^{18}$	3.15 × $10^{16}$
f14	**1.18 × $10^{15}$ **	1.07 × $10^{19}$	7.09 × $10^{16}$	2.18 × $10^{15}$	1.03 × $10^{19}$	6.92 × $10^{16}$
f15	1.10 × $10^{15}$	3.14 × $10^{18}$	3.07 × $10^{16}$	**1.09 × $10^{15}$ **	2.23 × $10^{18}$	3.11 × $10^{16}$

**Table 8:** Comparison results in similar hybrid algorithms.

	Adaptive RSA		CSARSA		CSHADE		SWEVOH-PSO		SWEVOH-CS		SWEVOH-BA	
Func.	Mean	Std	Mean	Std	Mean	Std	Mean	Std	Mean	Std	Mean	Std
f1	3.61 × $10^{14}$	1.14 × $10^{14}$	2.40 × $10^{14}$	3.45 × $10^{13}$	**1.11 × $10^{8}$ **	**1.24 × $10^{8}$ **	7.00 × $10^{10}$	8.30 × $10^{10}$	3.03 × $10^{10}$	7.81 × $10^{10}$	2.01 × $10^{11}$	3.04 × $10^{10}$
f2	7.96 × $10^{7}$	3.94 × $10^{7}$	5.47 × $10^{7}$	1.19 × $10^{7}$	1.41 × $10^{7}$	9.22 × $10^{5}$	6.05 × $10^{4}$	1.67 × $10^{4}$	**3.16 × $10^{4}$ **	1.97 × $10^{4}$	3.45 × $10^{4}$	**1.31 × $10^{4}$ **
f3	2.14 × $10^{4}$	1.89 × $10^{2}$	2.12 × $10^{4}$	1.05 × $10^{2}$	1.68 × $10^{4}$	6.52 × $10^{2}$	**2.14 × $10^{1}$ **	9.04 × $10^{-2}$	2.15 × $10^{1}$	5.61 × $10^{-2}$	2.16 × $10^{1}$	**1.39 × $10^{-2}$ **
f4	1.24 × $10^{17}$	9.29 × $10^{16}$	5.21 × $10^{16}$	3.28 × $10^{16}$	1.18 × $10^{14}$	1.26 × $10^{14}$	5.68 × $10^{12}$	3.64 × $10^{13}$	6.53 × $10^{12}$	4.63 × $10^{13}$	**5.33 × $10^{12}$ **	**6.67 × $10^{12}$ **
f5	8.63 × $10^{10}$	3.48 × $10^{10}$	5.80 × $10^{10}$	9.49 × $10^{9}$	2.05 × $10^{9}$	3.44 × $10^{8}$	4.08 × $10^{7}$	1.59 × $10^{7}$	1.82 × $10^{7}$	1.70 × $10^{7}$	**1.67 × $10^{7}$ **	**9.28 × $10^{6}$ **
f6	1.06 × $10^{9}$	1.29 × $10^{7}$	1.04 × $10^{9}$	9.18 × $10^{6}$	7.22 × $10^{7}$	3.01 × $10^{7}$	**1.05 × $10^{6}$ **	8.04 × $10^{3}$	1.06 × $10^{6}$	3.52 × $10^{3}$	1.06 × $10^{6}$	**2.21 × $10^{3}$ **
f7	1.87 × $10^{21}$	8.95 × $10^{21}$	4.11 × $10^{18}$	6.76 × $10^{18}$	**5.06 × $10^{10}$ **	**1.98 × $10^{11}$ **	9.51 × $10^{17}$	2.69 × $10^{19}$	1.13 × $10^{18}$	3.05 × $10^{19}$	5.19 × $10^{14}$	4.28 × $10^{15}$
f8	4.42 × $10^{21}$	4.72 × $10^{21}$	3.00 × $10^{21}$	2.47 × $10^{21}$	5.80 × $10^{18}$	4.12 × $10^{18}$	**2.60 × $10^{17}$ **	2.05 × $10^{18}$	2.94 × $10^{17}$	2.66 × $10^{18}$	2.71 × $10^{17}$	**3.87 × $10^{17}$ **
f9	1.03 × $10^{13}$	9.45 × $10^{12}$	5.32 × $10^{12}$	1.56 × $10^{12}$	2.09 × $10^{11}$	2.19 × $10^{10}$	3.44 × $10^{9}$	2.48 × $10^{9}$	1.58 × $10^{9}$	2.14 × $10^{9}$	**1.36 × $10^{9}$ **	**7.68 × $10^{8}$ **
f10	9.48 × $10^{10}$	7.66 × $10^{8}$	9.44 × $10^{10}$	6.81 × $10^{8}$	1.02 × $10^{9}$	4.34 × $10^{8}$	**9.39 × $10^{7}$ **	1.12 × $10^{6}$	9.44 × $10^{7}$	7.76 × $10^{5}$	9.47 × $10^{7}$	**5.10 × $10^{5}$ **
f11	7.29 × $10^{22}$	1.97 × $10^{23}$	1.70 × $10^{20}$	4.62 × $10^{20}$	**3.42 × $10^{10}$ **	**1.73 × $10^{10}$ **	4.19 × $10^{20}$	1.03 × $10^{22}$	7.77 × $10^{20}$	2.07 × $10^{22}$	4.30 × $10^{16}$	4.22 × $10^{17}$
f12	6.34 × $10^{15}$	3.36 × $10^{15}$	2.57 × $10^{15}$	6.39 × $10^{14}$	**8.07 × $10^{8}$ **	**2.33 × $10^{9}$ **	1.46 × $10^{12}$	2.03 × $10^{12}$	9.42 × $10^{11}$	1.58 × $10^{12}$	3.00 × $10^{12}$	8.30 × $10^{11}$
f13	3.58 × $10^{23}$	1.73 × $10^{24}$	3.58 × $10^{20}$	6.87 × $10^{20}$	**1.56 × $10^{11}$ **	**6.80 × $10^{11}$ **	3.84 × $10^{20}$	1.38 × $10^{22}$	3.34 × $10^{19}$	1.04 × $10^{21}$	3.15 × $10^{16}$	2.26 × $10^{17}$
f14	1.89 × $10^{22}$	4.07 × $10^{22}$	7.97 × $10^{20}$	1.82 × $10^{21}$	**1.56 × $10^{12}$ **	**8.43 × $10^{12}$ **	4.17 × $10^{20}$	1.64 × $10^{22}$	1.63 × $10^{20}$	3.93 × $10^{21}$	6.92 × $10^{16}$	5.20 × $10^{17}$
f15	2.50 × $10^{21}$	4.90 × $10^{21}$	9.04 × $10^{18}$	1.17 × $10^{19}$	**1.25 × $10^{10}$ **	**5.48 × $10^{9}$ **	2.07 × $10^{17}$	1.68 × $10^{18}$	1.49 × $10^{17}$	1.29 × $10^{18}$	3.11 × $10^{16}$	1.97 × $10^{17}$

**Table 9:** PSO *p*-values for function 1.

	SWEVOH-PSO	PSO
SWEVOH-PSO	×	SWS
PSO	7.01 × $10^{3}$	×

**Table 10:** PSO *p*-values for function 2.

	SWEVOH-PSO	PSO
SWEVOH-PSO	×	SWS
PSO	SWS	×

**Table 11:** PSO *p*-values for function 3.

	SWEVOH-PSO	PSO
SWEVOH-PSO	×	7.01 × $10^{3}$
PSO	SWS	×

**Table 12:** PSO *p*-values for function 4.

	SWEVOH-PSO	PSO
SWEVOH-PSO	×	1.86 × $10^{16}$
PSO	SWS	×

**Table 13:** PSO *p*-values for function 5.

	SWEVOH-PSO	PSO
SWEVOH-PSO	×	6.21 × $10^{4}$
PSO	SWS	×

**Table 14:** PSO *p*-values for function 6.

	SWEVOH-PSO	PSO
SWEVOH-PSO	×	1.67 × $10^{16}$
PSO	SWS	×

**Table 15:** PSO *p*-values for function 7.

	SWEVOH-PSO	PSO
SWEVOH-PSO	×	SWS
PSO	SWS	×

**Table 16:** PSO *p*-values for function 8.

	SWEVOH-PSO	PSO
SWEVOH-PSO	×	SWS
PSO	1.51 × $10^{15}$	×

**Table 17:** PSO *p*-values for function 9.

	SWEVOH-PSO	PSO
SWEVOH-PSO	×	5.93 × $10^{5}$
PSO	SWS	×

**Table 18:** PSO *p*-values for function 10.

	SWEVOH-PSO	PSO
SWEVOH-PSO	×	3.78 × $10^{10}$
PSO	SWS	×

**Table 19:** PSO *p*-values for function 11.

	SWEVOH-PSO	PSO
SWEVOH-PSO	×	SWS
PSO	SWS	×

**Table 20:** PSO *p*-values for function 12.

	SWEVOH-PSO	PSO
SWEVOH-PSO	×	SWS
PSO	6.97 × $10^{3}$	×

**Table 21:** PSO *p*-values for function 13.

	SWEVOH-PSO	PSO
SWEVOH-PSO	×	2.90 × $10^{16}$
PSO	SWS	×

**Table 22:** PSO *p*-values for function 14.

	SWEVOH-PSO	PSO
SWEVOH-PSO	×	SWS
PSO	SWS	×

**Table 23:** PSO *p*-values for function 15.

	SWEVOH-PSO	PSO
SWEVOH-PSO	×	SWS
PSO	8.49 × $10^{3}$	×

**Table 24:** CSA *p*-values for function 1.

	SWEVOH-CSA	CSA
SWEVOH-CSA	×	SWS
CSA	7.01 × $10^{-12}$	×

**Table 25:** CSA *p*-values for function 2.

	SWEVOH-CSA	CSA
SWEVOH-CSA	×	SWS
CSA	SWS	×

**Table 26:** CSA *p*-values for function 3.

	SWEVOH-CSA	CSA
SWEVOH-CSA	×	7.01 × $10^{-12}$
CSA	SWS	×

**Table 27:** CSA *p*-values for function 4.

	SWEVOH-CSA	CSA
SWEVOH-CSA	×	1.86 × $10^{-3}$
CSA	SWS	×

**Table 28:** CSA *p*-values for function 5.

	SWEVOH-CSA	CSA
SWEVOH-CSA	×	6.21 × $10^{-11}$
CSA	SWS	×

**Table 29:** CSA *p*-values for function 6.

	SWEVOH-CSA	CSA
SWEVOH-CSA	×	1.67 × $10^{-2}$
CSA	SWS	×

**Table 30:** CSA *p*-values for function 7.

	SWEVOH-CSA	CSA
SWEVOH-CSA	×	SWS
CSA	SWS	×

**Table 31:** CSA *p*-values for function 8.

	SWEVOH-CSA	CSA
SWEVOH-CSA	×	SWS
CSA	1.51 × $10^{-2}$	×

**Table 32:** CSA *p*-values for function 9.

	SWEVOH-CSA	CSA
SWEVOH-CSA	×	5.93 × $10^{-10}$
CSA	SWS	×

**Table 33:** CSA *p*-values for function 10.

	SWEVOH-CSA	CSA
SWEVOH-CSA	×	3.78 × $10^{-6}$
CSA	SWS	×

**Table 34:** CSA *p*-values for function 11.

	SWEVOH-CSA	CSA
SWEVOH-CSA	×	SWS
CSA	SWS	×

**Table 35:** CSA *p*-values for function 12.

	SWEVOH-CSA	CSA
SWEVOH-CSA	×	SWS
CSA	6.97 × $10^{-12}$	×

**Table 36:** CSA *p*-values for function 13.

	SWEVOH-CSA	CSA
SWEVOH-CSA	×	2.90 × $10^{-3}$
CSA	SWS	×

**Table 37:** CSA *p*-values for function 14.

	SWEVOH-CSA	CSA
SWEVOH-CSA	×	SWS
CSA	SWS	×

**Table 38:** CSA *p*-values for function 15.

	SWEVOH-CSA	CSA
SWEVOH-CSA	×	SWS
CSA	8.49 × $10^{-12}$	×

**Table 39:** BA *p*-values for function 1.

	SWEVOH-BA	BA
SWEVOH-BA	×	SWS
BA	SWS	×

**Table 40:** BA *p*-values for function 2.

	SWEVOH-BA	BA
SWEVOH-BA	×	1.48 × $10^{16}$
BA	SWS	×

**Table 41:** BA *p*-values for function 3.

	SWEVOH-BA	BA
SWEVOH-BA	×	7.43 × $10^{-15}$
BA	SWS	×

**Table 42:** BA *p*-values for function 4.

	SWEVOH-BA	BA
SWEVOH-BA	×	3.58 × $10^{15}$
BA	SWS	×

**Table 43:** BA *p*-values for function 5.

	SWEVOH-BA	BA
SWEVOH-BA	×	SWS
BA	SWS	×

**Table 44:** BA *p*-values for function 6.

	SWEVOH-BA	BA
SWEVOH-BA	×	4.21 × $10^{14}$
BA	SWS	×

**Table 45:** BA *p*-values for function 7.

	SWEVOH-BA	BA
SWEVOH-BA	×	2.96 × $10^{16}$
BA	SWS	×

**Table 46:** BA *p*-values for function 8.

	SWEVOH-BA	BA
SWEVOH-BA	×	SWS
BA	SWS	×

**Table 47:** BA *p*-values for function 9.

	SWEVOH-BA	BA
SWEVOH-BA	×	4.56 × $10^{15}$
BA	SWS	×

**Table 48:** BA *p*-values for function 10.

	SWEVOH-BA	BA
SWEVOH-BA	×	2.67 × $10^{16}$
BA	SWS	×

**Table 49:** BA *p*-values for function 11.

	SWEVOH-BA	BA
SWEVOH-BA	×	SWS
BA	SWS	×

**Table 50:** BA *p*-values for function 12.

	SWEVOH-BA	BA
SWEVOH-BA	×	SWS
BA	SWS	×

**Table 51:** BA *p*-values for function 13.

	SWEVOH-BA	BA
SWEVOH-BA	×	2.36 × $10^{16}$
BA	SWS	×

**Table 52:** BA *p*-values for function 14.

	SWEVOH-BA	BA
SWEVOH-BA	×	SWS
BA	SWS	×

**Table 53:** BA *p*-values for function 15.

	SWEVOH-BA	BA
SWEVOH-BA	×	SWS
BA	SWS	×

## Data Availability

Data is available on http://doi.org/10.6084/m9.figshare.24899883 (accessed on 19 December 2023).
